# Assessing and predicting type 2 diabetes risk with triglyceride glucose‐body mass index in the Chinese nondiabetic population—Data from long‐term follow‐up of Da Qing IGT and Diabetes Study

**DOI:** 10.1111/1753-0407.70001

**Published:** 2024-10-04

**Authors:** Haixu Wang, Siyao He, Jinping Wang, Xin Qian, Bo Zhang, Zhiwei Yang, Bo Chen, Guangwei Li, Qiuhong Gong, for the Da Qing Diabetes Prevention Outcome Study Group

**Affiliations:** ^1^ Center of Endocrinology, National Center of Cardiology &Fuwai Hospital Chinese Academy of Medical Sciences and Peking Union Medical College Beijing China; ^2^ Department of Cardiology Da Qing Oilfield General Hospital Da Qing China; ^3^ Department of Endocrinology China‐Japan Friendship Hospital Beijing China; ^4^ Division of Non‐Communicable Disease Control and Community Health Chinese Center for Disease Control and Prevention Beijing China

**Keywords:** Da Qing Study, predictability, triglyceride glucose‐body mass index, type 2 diabetes

## Abstract

**Aims:**

We intended to characterize the superiority of triglyceride glucose‐body mass index (TyG‐BMI) in predicting type 2 diabetes mellitus (T2DM) compared with triglyceride glucose (TyG) and homeostatic model assessment for insulin resistance (HOMA‐IR).

**Methods:**

A total of 699 nondiabetic participants in the Da Qing IGT and Diabetes Study were involved in the present analysis and classified according to the median of baseline TyG‐BMI, namely the G1 (low TyG‐BMI) and G2 (high TyG‐BMI) groups. Information on developing diabetes was assessed from 1986 to 2020.

**Results:**

During the 34‐year follow‐up, after adjustment for confounders, the G2 group had a higher risk of developing type 2 diabetes than the G1 group (hazard ratio [HR]: 1.92, 95% confidence interval [CI]: 1.51–2.45, *p* < 0.0001). Restricted cubic spline analyses showed that increased TyG‐BMI was linearly related to higher risks of type 2 diabetes (*p* for non‐linearity>0.05). Time‐dependent receiver operator characteristics curves suggested that TyG‐BMI exhibited higher predictive ability than TyG (6‐year: area under the curve [AUC]_TyG‐BMI_ vs. AUC_TyG_, 0.78 vs. 0.70, *p* = 0.03; 34‐year: AUC_TyG‐BMI_ vs. AUC_TyG_, 0.79 vs. 0.73, *p* = 0.04) and HOMA‐IR (6‐year: AUC_TyG‐BMI_ vs. AUC_HOMA‐IR_, 0.78 vs. 0.70, *p* = 0.07; 34‐year: AUC_TyG‐BMI_ vs. AUC_HOMA‐IR_, 0.79 vs. 0.71, *p* = 0.04) in both short and long terms, and the thresholds of TyG‐BMI to predict type 2 diabetes were relatively stable (195.24–208.41) over the 34‐year follow‐up.

**Conclusions:**

In this post hoc study, higher TyG‐BMI was associated with an increased risk of type 2 diabetes and demonstrated better predictability than TyG and HOMA‐IR, favoring the application of TyG‐BMI as a potential tool for evaluating the risk of type 2 diabetes in clinical practice.

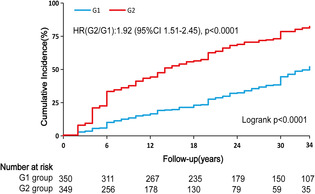

## Graphical abstract

Highlights

- After the 34‐year follow‐up, increased TyG‐BMI was independently associated with a higher risk of type 2 diabetes.
- TyG‐BMI exhibited higher predictive ability than TyG or HOMA‐IR in both short (6‐year) and long terms (34‐year). The best thresholds of TyG‐BMI to predict diabetes were relatively stable (195.24–208.41).
- Our results provide the possibility to utilize TyG‐BMI for predicting diabetes risk in clinical practice.

## 1 INTRODUCTION

The number of patients with diabetes is rising in the world. Based on the International Diabetes Federation Diabetes Atlas Ninth Edition, the prevalence will rise from 463 million in 2019 to 700 million by 2045.^1^ Moreover, the associated complications have imposed large health and social burdens. Therefore, early identification of high‐risk people may prove to be highly beneficial. Insulin resistance (IR) is the major pathophysiologic factor of type 2 diabetes, and the current gold standard for IR, the hyperinsulinemic‐euglycemic clamp (HEC)^2^ is time‐consuming, expensive and hard in practical scenarios. Hence, a more convenient method for predicting diabetes is needed.

Traditionally, the homeostasis model assessment of insulin resistance (HOMA‐IR) has been applied in a wide range of clinical and academic settings to estimate IR. Triglyceride (TG) glucose (TyG), consisting of TG and fasting plasma glucose (FPG), also exhibited a high value in assessing diabetes risk.^3^ In recent years, triglyceride glucose‐body mass index (TyG‐BMI), was suggested to be a better predictor for IR than TyG alone in several studies.^4^, ^5^ Song et al exhibited that compared to subjects in the lowest quartile, those in the highest quartile of TyG‐BMI were associated with a 2.28‐fold increased risk of diabetes. Of note, it has also been reported that TyG‐BMI owned an excellent predictive value than TyG.^6^

In the present study, we did post hoc analysis in nondiabetic participants in Da Qing IGT and Diabetes Study over the 34‐year follow‐up, to clarify whether or not TyG‐BMI has superiority compared with TyG and HOMA‐IR in predicting the occurrence of type 2 diabetes.

## 2 MATERIALS AND METHODS

### 2.1 Study population

The design and population features of our research have been described in detail.^7^, ^8^ In short, 110 660 individuals were enrolled in Daqing, China in 1986, and an oral glucose tolerance test (OGTT) was conducted to determine diabetes status according to 1985 World Health Organization (WHO) criteria. In total, 576 had impaired glucose tolerance (IGT), and age‐ and sex‐matched 519 individuals with normal glucose tolerance (NGT) were chosen for comparison. Active lifestyle intervention lasted for 6 years among the IGT group, then participants were kept under medical care from usual providers. We performed the 20‐, 30‐, and 34‐year follow‐up study to investigate the incidence of type 2 diabetes in the original participants in 2006, 2016, and 2020. Finally, 699 nondiabetic people with complete data on TG and FPG at baseline were involved in the present analysis (Figure S1).

### 2.2 Data acquisition

Data collection was conducted by trained staff through clinical examinations, personal interviews, and medical record reviews. If participants were unable to visit the hospital (e.g., living outside Daqing city or having poor physical function), they would be examined at home, interviewed by telephone, and then examined in a local hospital. Diabetes status was defined by 75 g OGTT results according to 1985 WHO criteria at the follow‐up examinations in 1992, 2006, 2016, and 2020 if participants had not been diagnosed previously, self‐reported diagnosis in personal interviews, or evidence of increased blood glucose or use of hypoglycemic agents in medical record reviews. When disagreement persisted between the physicians, an independent senior physician was consulted to arbitrate.

Formulas used to calculate the metrics: BMI = weight (kg)/height (m^2^); TyG = ln [FPG (mg/dL) × TG (mg/dL) × 0.5]; TyG‐BMI = BMI × TyG; HOMA‐IR = fasting insulin (mU/L) × FPG (mmol/L)/22.5. Participants were classified into two groups based on the median of baseline TyG‐BMI, sequentially labeled as the G1 (low TyG‐BMI) and G2 (high TyG‐BMI) groups.

### 2.3 Statistical analysis

Continuous data were presented as mean ± standard error, and categorical data were presented as number (percentage). Group differences in baseline characteristics were evaluated using analysis of variance (ANOVA) or chi‐square test. The incidence rate of diabetes was estimated by dividing the number of diabetes by the total person‐years of observation. Data on total cholesterol (TC) were only available for 616 participants (88.13%). Hence, multiple imputation was performed using PROC MI in SAS statistical software version 9.4 (SAS Institute) to impute missing values, and the resulting estimates were averaged. The time to follow‐up began from randomization (1986) to the date on which participants were diagnosed with diabetes, or December 31, 2020, for those without diabetes, or the last visit for those who are lost to follow‐up. Cumulative incidence was computed using Kaplan–Meier curves. Cox regression models were performed to evaluate relationships between TyG‐BMI and diabetes risk after adjustment for age, sex, smoking, systolic blood pressure (SBP), TC, and lifestyle intervention. Restricted cubic splines (RCS) analysis was performed to explore dose–response association. We further performed subgroup analyses according to age, sex, smoking, and glucose regulation status as sensitivity analyses and evaluated the potential interaction effect. To evaluate and compare abilities to predict diabetes during 34‐year follow‐up among TyG‐BMI, TyG, and HOMA‐IR, time‐dependent receiver operating characteristic (ROC) analysis was performed to calculate and compare the area under the curve (AUC) and the prediction thresholds at each time point.

Two‐tailed *p* values <0.05 indicated statistical significance. Analyses were conducted in SAS software (version 9.4; SAS Institute, Cary, NC) and R software (version R 4.2.2).

## 3 RESULTS

### 3.1 Baseline characteristics

Table 1 presents the demographic and metabolic features at baseline. A total of 699 nondiabetic subjects were included in the present study, of which 395 subjects (56.51%) were classified into the NGT group and 304 (43.49%) into the IGT group. The median of TyG‐BMI was 207.7. Among the enrolled subjects, 52.5% were male, 47.5% were female, and their mean age was 44.7 years old. Participants with higher TyG‐BMI tend to be older and had higher BMI, blood pressure, glucose level, blood lipid, insulin level, and insulin resistance (all *p* < 0.05).

**TABLE 1:** Baseline characteristics of participants in groups with low TyG‐BMI (G1) and high TyG‐BMI (G2).

	All (*n* = 699)	G1 (*n* = 350) (TyG‐BMI < 207.7)	G2 (*n* = 349) (TyG‐BMI ≥ 207.7)	*p* value^a^
Age (years)	44.67 ± 0.34	43.70 ± 0.51	45.64 ± 0.45	0.005
Sex, *n* (%)				0.52
Men	367 (52.50%)	188 (53.71%)	179 (51.29%)	
Women	332 (47.50%)	162 (46.29%)	170 (48.71%)	
Smoking, *n* (%)	302 (43.20%)	169 (48.29%)	133 (38.11%)	0.007
BMI (kg/m^2^)	24.55 ± 0.14	21.78 ± 0.11	27.32 ± 0.13	<0.0001
FPG (mmol/L)	5.07 ± 0.03	4.81 ± 0.03	5.32 ± 0.05	<0.0001
2hPG(mmol/L)	6.72 ± 0.08	6.04 ± 0.10	7.41 ± 0.12	<0.0001
FINS (mU/L)	12.90 ± 0.70	9.43 ± 0.75	17.14 ± 1.20	<0.0001
1hINS (mU/L)	65.92 ± 3.10	46.22 ± 3.28	89.32 ± 5.20	<0.0001
2hINS (mU/L)	58.18 ± 3.17	40.88 ± 3.38	78.80 ± 5.41	<0.0001
SBP (mmHg)	127.22 ± 0.87	118.03 ± 0.89	136.43 ± 1.32	<0.0001
DBP (mmHg)	84.59 ± 0.54	79.06 ± 0.55	90.13 ± 0.82	<0.0001
TC (mmol/L)	4.95 ± 0.05	4.72 ± 0.06	5.20 ± 0.08	<0.0001
TG (mmol/L)	1.52 ± 0.05	0.98 ± 0.02	2.06 ± 0.09	<0.0001
HOMA‐IR	2.98 ± 0.17	2.06 ± 0.18	4.11 ± 0.30	<0.0001
Lifestyle intervention, *n* (%)	247 (35.34%)	81 (23.14%)	166 (47.56%)	<0.0001

*Note*: Continuous data were presented as mean ± standard error, and categorical data were presented as number (percentage). Groups were classified by the median of triglyceride glucose‐body mass index (TyG‐BMI) at baseline, TyG‐BMI < 207.7 was the G1 group, and TyG‐BMI ≥ 207.7 was the G2 group.

Abbreviations: 1hINS, 1‐h insulin level; 2hINS, 2‐h insulin level; 2hPG, 2‐h plasma glucose; BMI, body mass index; DBP, diastolic blood pressure; FINS, fasting insulin level; FPG, fasting plasma glucose; HOMA‐IR, homeostatic model assessment for insulin resistance; SBP, systolic blood pressure; TC, total cholesterol; TG, triglyceride; TyG, triglyceride glucose.

^a^ *p* values were referred to the difference between the G1 and G2 groups.

### 3.2 TyG‐BMI and the risk of type 2 diabetes

**FIGURE 1:**
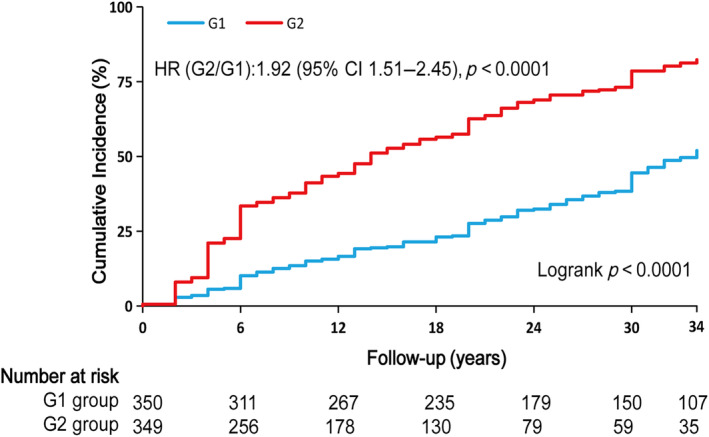
Cumulative incidence of newly diagnosed diabetes in groups with different triglyceride glucose‐body mass index (TyG‐BMI) over the 34‐year follow‐up. HR referred to the hazard ratio in the high TyG‐BMI (G2) group compared with the low TyG‐BMI (G1) group, which were adjusted by age, sex, smoking, systolic blood pressure, total cholesterol, and lifestyle intervention. CI, confidence interval.

**FIGURE 2:**
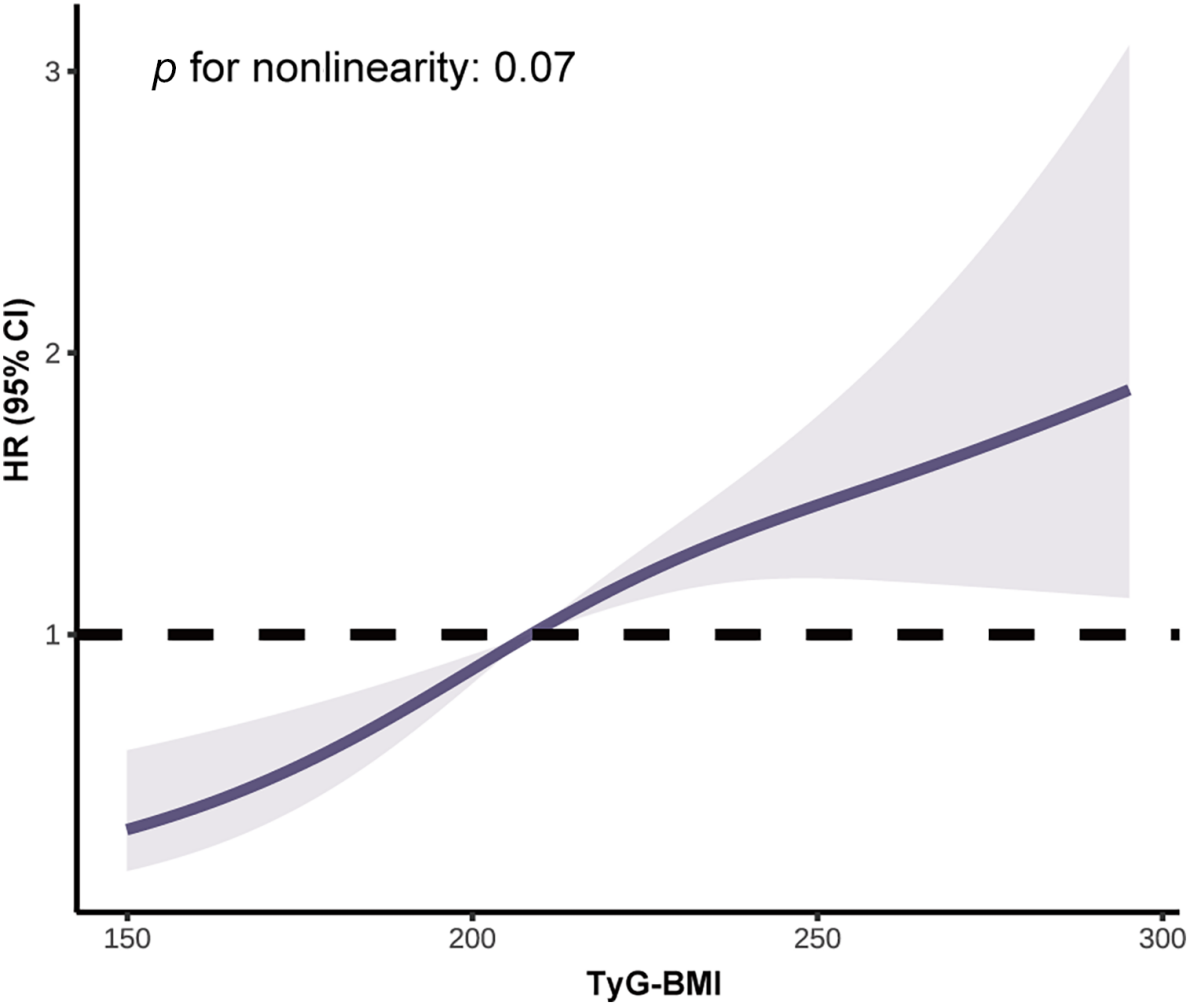
Dose–response relationships between triglyceride glucose‐body mass index (TyG‐BMI) and diabetes risk. The model was adjusted for age, sex, systolic blood pressure, smoking, total cholesterol, and lifestyle intervention. CI, confidence interval; HR, hazard ratio.

**FIGURE 3:**
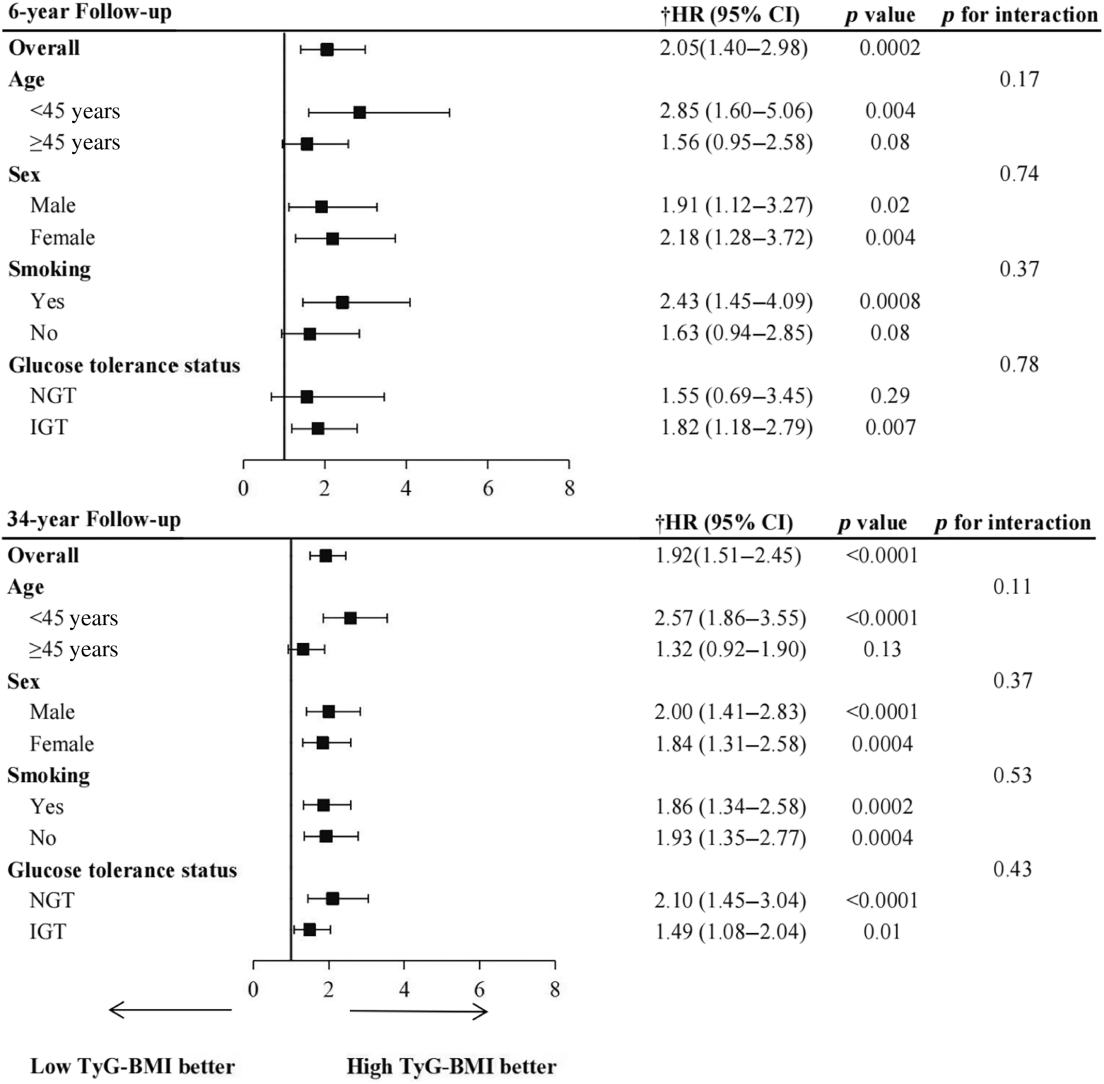
Forest plot of diabetes risk associated with triglyceride glucose‐body mass index (TyG‐BMI) at 6‐year and 34‐year follow‐up. †Hazard ratios (HRs) were adjusted for age, sex, systolic blood pressure, smoking, total cholesterol, and lifestyle intervention. CI, confidence interval; IGT, impaired glucose tolerance; NGT, normal glucose tolerance.

Table 2 showed the incidence rates and cumulative incidence of type 2 diabetes during the 34‐year follow‐up. The rates for diabetes were 18.96 (95% confidence interval [CI]: 16.02–22.28) and 48.75 (95% CI: 42.87–55.21) per 1000 person‐years in the G1 and G2 groups, respectively. During the 34‐year follow‐up, the cumulative incidence in the G2 group was high (82.3%, 95% CI: 76.9–86.6), whereas the G1 group was low (52.0%, 95% CI: 45.7–57.9) (Figure 1). After adjusting for age, sex, smoking, SBP, TC, and lifestyle intervention, diabetes risk was higher in the G2 group (HR: 1.92, 95% CI: 1.51–2.45, *p* < 0.0001) than the G1 group. RCS analysis confirmed a linear relationship between TyG‐BMI and diabetes risk (Figure 2). Additionally, we validated the results in subgroups and found that they were stable in all the subgroups (Figure 3) without interaction of specific subgroup analysis (all *p* > 0.05).

**TABLE 2:** Cumulative incidence and incidence rate in groups with low TyG‐BMI (G1) and high TyG‐BMI (G2) at 34‐year follow‐up.

	G1	G2	HR^a^ (95% CI)	*p* value
Cases/person‐years	147/7754	248/5087		
Cumulative incidence (%, 95% CI)	51.97 (45.69–57.88)	82.33 (76.88–86.60)		
Cases per 1000 person‐years (95% CI)	18.96 (16.02–22.28)	48.75 (42.87–55.21)	1.92 (1.51–2.45)	<0.0001

Abbreviations: CI, confidence interval; HR, hazard ratio; TyG‐BMI, triglyceride glucose‐body mass index.

^a^ HR referred to the hazard ratio in the G2 group compared with the G1 group, which were adjusted by age, sex, smoking, systolic blood pressure, total cholesterol, and lifestyle intervention. Groups were classified by the median of TyG‐BMI at baseline, TyG‐BMI < 207.7 was the G1 group, and TyG‐BMI ≥ 207.7 was the G2 group.

### 3.3 Predictive performance of TyG‐BMI, TyG, and HOMA‐IR for type 2 diabetes

**FIGURE 4:**
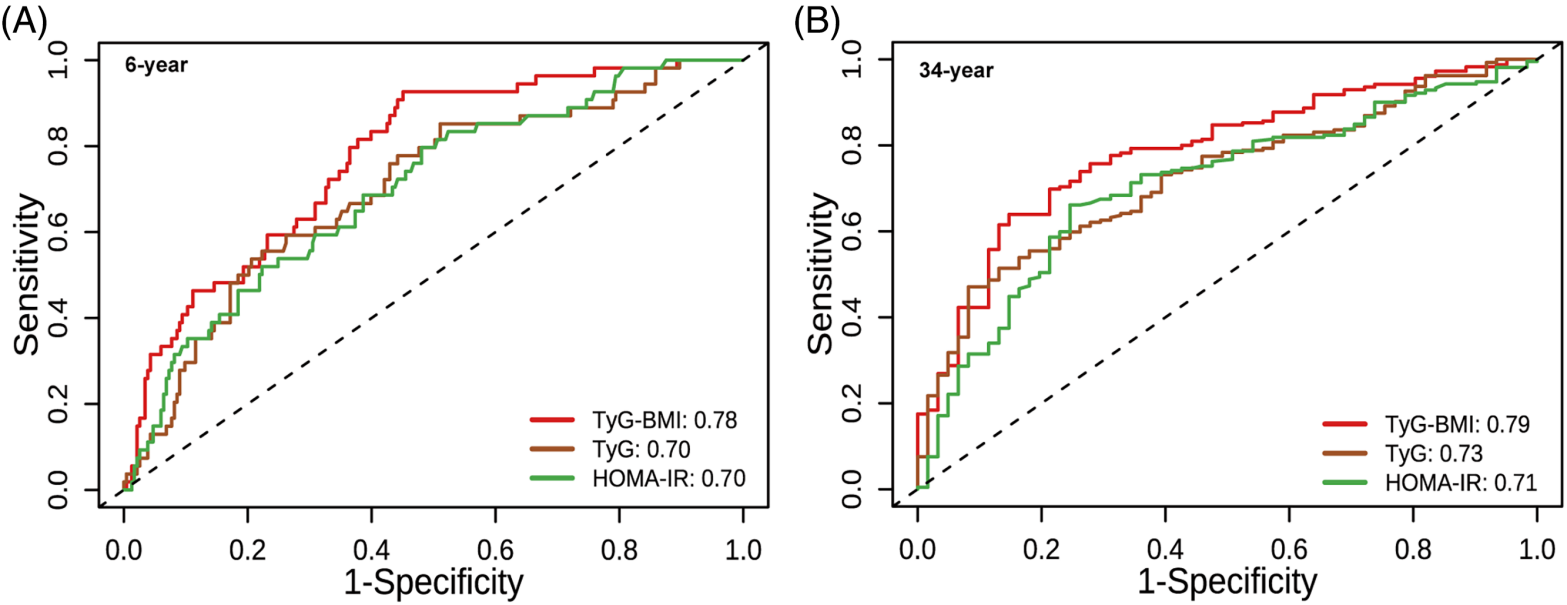
Time‐dependent receiver operating characteristic curves (ROCs) for different parameters in predicting diabetes at 6‐year (A) and 34‐year (B) follow‐up. HOMA‐IR, homeostatic model assessment for insulin resistance; TyG, triglyceride glucose; TyG‐BMI, triglyceride glucose‐body mass index.

To evaluate the performance of TyG‐BMI, TyG, and HOMA‐IR in predicting the incidence of type 2 diabetes, time‐dependent ROC analysis at 6‐year follow‐up (Figure 4A) and 34‐year follow‐up (Figure 4B) was performed. The AUC of TyG‐BMI reached 0.78 (95% CI: 0.71–0.84) at 6‐year follow‐up and 0.79 (95% CI: 0.72–0.85) at 34‐year follow‐up. To observe how their predictive power has changed over time, the AUCs at different time points were summarized in Table 3. Overall, all the indices had good predictive value for diabetes, demonstrating a stable trend over time. Furthermore, the AUCs of TyG‐BMI were slightly higher than TyG and HOMA‐IR, indicating that TyG‐BMI was a superior predictor for both short‐term (6‐year: AUC_TyG‐BMI_ vs. AUC_TyG_, 0.78 vs. 0.70, *p* = 0.03; AUC_TyG‐BMI_ vs. AUC_HOMA‐IR_, 0.78 vs. 0.70, *p* = 0.07; AUC_TyG_ vs. AUC_HOMA‐IR_, 0.70 vs. 0.70, *p* = 0.97) and long‐term onset diabetes (34‐year: AUC_TyG‐BMI_ vs. AUC_TyG_, 0.79 vs. 0.73, *p* = 0.04; AUC_TyG‐BMI_ vs. AUC_HOMA‐IR_, 0.79 vs. 0.71, *p* = 0.04; AUC_TyG_ vs. AUC_HOMA‐IR_, 0.73 vs. 0.71, *p* = 0.68).

**TABLE 3:** AUC and the thresholds for TyG‐BMI, TyG, and HOMA‐IR in predicting diabetes over the 34‐year follow‐up.

Predicting time	6‐year	20‐year	30‐year	34‐year
AUC				
TyG‐BMI	0.78	0.74	0.82	0.79
TyG	0.70	0.70	0.77	0.73
HOMA‐IR	0.70	0.70	0.75	0.71
Cutoff points				
TyG‐BMI	203.01	202.27	195.24	208.41
TyG	8.44	8.73	8.68	8.73
HOMA‐IR	3.40	3.85	3.48	3.48

*Note*: Based on the results of the time‐dependent receiver operating characteristic curve test.

Abbreviations: AUC, area under the curves; HOMA‐IR, homeostatic model assessment for insulin resistance; TyG, triglyceride glucose; TyG‐BMI, triglyceride glucose‐body mass index.

To predict diabetes risk at 6, 20, 30, and 34‐year follow‐up, we calculated the best thresholds of the above indicators (Table 3). When TyG‐BMI was higher than 203.0, there was an increased risk of people developing diabetes over the 6‐year interval. Similarly, a TyG‐BMI level of more than 208.4 was associated with higher diabetes risk over the 34‐year follow‐up. In addition, the best thresholds of TyG‐BMI during 34‐year follow‐up were relatively stable (TyG‐BMI: 195.24–208.41). Prediction thresholds of TyG and HOMA‐IR also fluctuated within a narrow range (TyG: 8.44–8.73; HOMA‐IR: 3.40–3.85).

## 4 DISCUSSION

Our study investigated the relationship between TyG‐BMI and diabetes incidence in Chinese nondiabetic population over the 34‐year follow‐up. After adjusting for confounders including age, sex, smoking, SBP, TC, and lifestyle intervention, when compared with the G1 group, G2 group was associated with a 92% increased risk of type 2 diabetes. Our study also observed a positive linear correlation between TyG‐BMI and diabetes risk. A comparison of predictive accuracy between TyG‐BMI, TyG, and HOMA‐IR was further conducted at different time points. Compared with TyG and HOMA‐IR, TyG‐BMI was of the highest value for predicting both short‐term and long‐term risk of diabetes. Of note, the best thresholds of TyG‐BMI for predicting diabetes fluctuated little, demonstrating the feasibility of TyG‐BMI as a stable predictor.

Our study extended the literature in several ways. First, elevated TyG‐BMI was related to increased diabetes risk in Chinese people over a long period. First proposed in 2016,^5^ TyG‐BMI has also been suggested as an alternative index of IR.^5^ A few cross‐sectional^9^, ^10^ and prospective studies^6^, ^11^ also presented a positive correlation between TyG‐BMI and diabetes risk. Data from a cohort study demonstrated that G2, defined as the highest quintile, was responsible for a 4.65‐fold increased risk of developing diabetes after 3.1‐years follow‐up.^11^ Similar results were also reported among Japanese with normal glycemic levels. However, such an association may not be generalized due to the short follow‐up period. After a 34‐year follow‐up, we found that TyG‐BMI was independently correlated with an increased risk of developing diabetes in Chinese population.

Second, we demonstrated the superiority of TyG‐BMI for diabetes prediction compared with TyG or HOMA‐IR. TyG‐BMI is a combined parameter of TyG and obesity indices. The latest study suggested that TyG‐related parameters provided better identification of fatty liver^12^ and prediction of coronary artery calcification progression^13^ than TyG alone. Researchers are increasingly exploring the possibility that TyG combined with obesity indices can improve the risk assessment and prediction for diabetes, but the results remain inconsistent.^5^, ^14^, ^15^, ^16^ A cross‐sectional study among the Chinese elderly population showed that TyG‐BMI failed to enhance the value in evaluating the occurrence of diabetes,^14^ while a cohort study demonstrated that, in the Chinese middle‐aged and elderly population, TyG‐BMI was more strongly correlated with diabetes risk.^15^ After analyzing retrospective data from 5575 Chinese participants and with a 3‐year follow‐up, Xing et al. found that TyG was a more effective predictor of DM than TyG‐BMI.^17^ Of note, men accounted for 79.3%. Differences in conclusions may in part be attributed to sex disparities in the risk factors for diabetes. Indeed, associations between BMI and diabetes risk were generally stronger in women than in men.^18^ Regretfully, because of our small sample size, it is difficult to further compare the predictive value of TyG and TyG‐BMI in all‐female or all‐male groups. Another reason for the inconsistent conclusions might be the long‐term follow‐up in our study (34 years). It is possible that a longer follow‐up time allowed for the occurrence of diabetes, which contributed to greater certainty for our results. As TyG‐BMI additionally reflected the role obesity plays in diabetes development,^19^ we speculated that the predictive performance of TyG‐BMI would be better than TyG, which was subsequently supported by our results.

Third, TyG‐BMI was more accurate than HOMA‐IR in predicting diabetes. The association between HOMA‐IR or TyG‐BMI and diabetes risk has been reported in many studies,^6^, ^20^ but comparative studies on the two parameters for predicting future diabetes are extremely limited. Our results supported that TyG‐BMI performed better than HOMA‐IR in predicting incident diabetes.

Fourth, the superior performance of TyG‐BMI might be explained by the effects of blood glucose, lipid metabolism, and obesity on insulin sensitivity.^21^ The presence of chronic hyperglycemia has been demonstrated to increase oxidative stress in the pancreatic islets,^22^ thus causing a continuous decline in β‐cell function and IR.^23^ On the other hand, excessive TG can lead to ectopic fat deposition in muscle cells^24^ and pancreatic islet cells, thus impairing islet β‐cell function.^25^ Also, some studies showed that obesity was an important predictor for new‐onset diabetes.^26^ The underlying mechanisms may be related to the dysregulation of adipocytokines and other active molecules produced by adipocytes, which were proved to increase with higher adiposity accumulation.^27^

Fifth, our findings may also have implications for diabetes screening in the general population. As the HEC test is not easily applicable for large‐scale assessment purposes, an increasing number of parameters have been recommended for diabetes prediction. However, there is a lack of consensus regarding which indices work best. By comparing TyG‐BMI with the most common indices, our results suggested that TyG‐BMI had the best predictive performance for the occurrence of diabetes. As TyG‐BMI, a parameter positively associated with diabetes risk, only requires a single blood drawing and height and weight measurement, it might be used as an effective and economical way to identify high‐risk individuals of diabetes in primary health care, thus reducing disease burden.

Our research has its advantages. First, OGTT results at baseline and during follow‐up were accessible, offering enough information to confirm the presence or absence of diabetes. Second, the subjects seldom migrated; thus, the rate of loss to follow‐up was relatively low. Third, the sufficiently long follow‐up period allowed us to discover the development of diabetes. Fourth, our present study is one of the few studies to longitudinally compare TyG‐BMI with TyG and HOMA‐IR as predictors of diabetes risk, which offers further evidence for the advantages of TyG‐BMI.

Our research also has the following defects. First, regular systematic examinations were not conducted during follow‐up periodically. Diabetes status was assessed in some participants by self‐report or use of diabetic medications, rather than by OGTTs, which may have resulted in underestimation of diabetes incidence. However, our previous study had suggested that people at high risk for diabetes had already converted into diabetes during the 6‐year active intervention period, leaving a low‐risk group to convert at a slower rate during the extended follow‐up.^28^ Considering the incidence of diabetes during the long‐term follow‐up period was significantly reduced, we speculated the impact due to such bias was minimal. Second, the sample size was relatively small, and prospective studies with larger populations would be needed to validate and generalize the conclusions. Third, post hoc analyses are likely to lead to biased conclusions, asking for further investigation and replication. Fourth, lifestyle intervention conducted in IGT participants probably reduced the actual incidence of diabetes. Therefore, to minimize this effect, we adjusted for lifestyle intervention in our regression models. Fifth, because of a lack of data on waist circumference, other TyG index‐related parameters, such as triglyceride glucose‐waist circumference (TyG‐WC) and triglyceride glucose‐waist height ratio (TyG‐WHtR), were not studied. Sixth, all participants were Chinese, and our conclusion may not be generalizable to other populations.

## 5 CONCLUSION

Over the 34‐year follow‐up, TyG‐BMI was independently associated with the risk of type 2 diabetes in Chinese people. Compared with TyG and HOMA‐IR, TyG‐BMI was the most reliable marker for predicting diabetes. Perhaps, more emphasis may need to be placed on its role as a clinical indicator of diabetes risk to improve the identification of high‐risk subjects and facilitate the prevention of diabetes.

## AUTHOR CONTRIBUTIONS

Q.G. contributed to the conception and design of the work, data collection and analysis, and reviewing and editing of the final report. H.W. was involved in data analysis, drafting, preparation, and revising the manuscript. S.H. participated in data collection and statistical analysis. J.W. and X.Q. were involved in data collection, B.Z., Z.Y., and B.C. were involved in data collection and management, and G.L. supervised the study and provided critical review and revisions. All authors gave the final approval of the version published. G.L. is the guarantor of this work and, as such, has full access to all the data in the study and takes responsibility for the integrity of the data and the accuracy of the data analysis.

## CONFLICT OF INTEREST STATEMENT

The authors declare no conflicts of interest.

## PATIENT CONSENT STATEMENT

All study participants, or proxies who served as informants for the deceased, provided written informed consent.

## PERMISSION TO REPRODUCE MATERIAL FROM OTHER SOURCES

This study did not include data that required permission to reproduce material from other sources.

## CLINICAL TRIAL REGISTRATION

Da Qing IGT and Diabetes Study began in 1986 and the intensive intervention phase ended in 1992. The present analysis was a post hoc analysis and therefore has not been registered.

## Supporting information


**Figure S1.** Flow chart.

## Data Availability

All data used to support the findings of this research are available on reasonable request to the corresponding author and subject to an approved proposal and data access agreement.
