# Supplementary material for: Assessing and predicting type 2 diabetes risk with triglyceride glucose‐body mass index in the Chinese nondiabetic population—Data from long‐term follow‐up of Da Qing IGT and Diabetes Study
Source: J Diabetes. 2024 Oct 4;16(10):e70001. doi: 10.1111/1753-0407.70001 (PMC11450669; doi:10.1111/1753-0407.70001)
Supplement: Supplementary file 1 — Figure S1. Flow chart. [file JDB-16-e70001-s001.docx]

Supplementary material

Figure S1. Flow chart


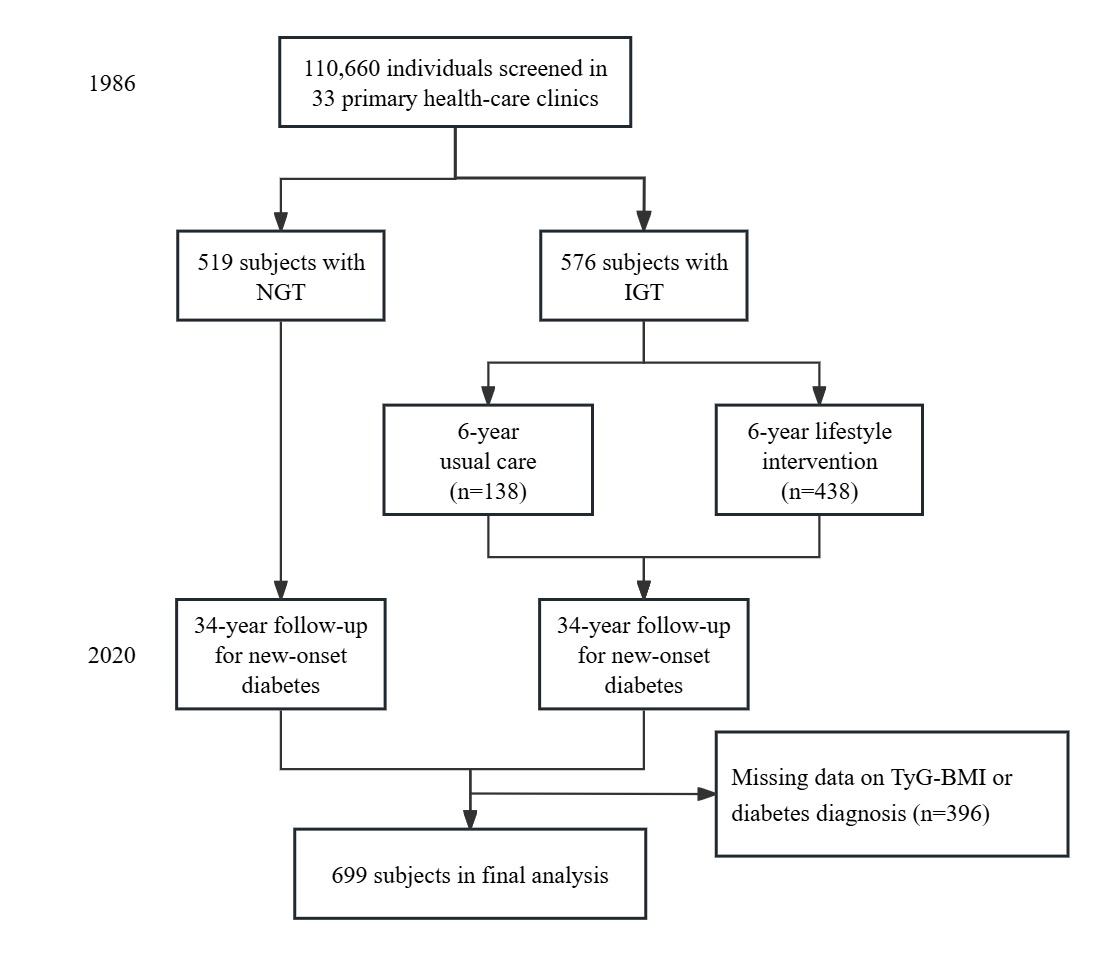


Abbreviations: TyG-BMI, triglyceride glucose-body mass index; NGT, normal glucose tolerance; IGT, impaired glucose tolerance.
